# Expression of posterior Hox genes and opisthosomal appendage development in a mygalomorph spider

**DOI:** 10.1007/s00427-023-00707-9

**Published:** 2023-07-27

**Authors:** Ralf Janssen, Matthias Pechmann

**Affiliations:** 1 Department of Earth Sciences, Palaeobiology, Uppsala University, Villavägen 16, 75236 Uppsala, Sweden; 2 Institute for Zoology, Biocenter, University of Cologne, Zuelpicher Str. 47b, 50674 Cologne, Germany

**Keywords:** Spider development, Spinneret, Book lung, Trachea, Genitalia, Tarantula

## Abstract

Spiders represent an evolutionary successful group of chelicerate arthropods. The body of spiders is subdivided into two regions (tagmata). The anterior tagma, the prosoma, bears the head appendages and four pairs of walking legs. The segments of the posterior tagma, the opisthosoma, either lost their appendages during the course of evolution or their appendages were substantially modified to fulfill new tasks such as reproduction, gas exchange, and silk production. Previous work has shown that the homeotic Hox genes are involved in shaping the posterior appendages of spiders. In this paper, we investigate the expression of the posterior Hox genes in a tarantula that possesses some key differences of posterior appendages compared to true spiders, such as the lack of the anterior pair of spinnerets and a second set of book lungs instead of trachea. Based on the observed differences in posterior Hox gene expression in true spiders and tarantulas, we argue that subtle changes in the Hox gene expression of the Hox genes *abdA* and *AbdB* are possibly responsible for at least some of the morphological differences seen in true spiders *versus* tarantulas.

**Supplementary Information:**

The online version contains supplementary material available at 10.1007/s00427-023-00707-9.

## Introduction

Arthropods represent the most dominant group of animals in terms of both the number of individuals and the number of species. The main reason for their evolutionary success is likely their morphological diversity, a feature that is correlated with the segmented nature of the arthropod main body axis (e.g., Budd 2001). In the last common ancestor of arthropods, each body segment carried one pair of appendages, which were very similar to each other, if not even the same on each segment. Later during evolution, however, more and more anterior segments became specialized for the perception of the environment, food gathering and processing, and specialized ways of locomotion (reviewed in Jockusch 2017, Ortega-Hernández et al. 2017). Other segments, often the most posterior ones, frequently lost their appendages, or their appendages became heavily modified. In parallel with these processes, segments carrying similar specialized appendages formed functional body units (tagmata), like the head, the thorax, and the abdomen of insects.

The genetics of arthropod body segmentation, tagmosis, and appendage development are best studied in the vinegar fly *Drosophila melanogaster* in which most of the anterior-posterior body axis (AP axis) is subdivided into segments by a hierarchic segmentation gene cascade (Nüsslein-Volhard and Wieschaus 1980, reviewed in Klingler and Tautz 1999, Clark et al. 2019). At the bottom of this cascade, homeotic selector genes (including the famous Hox genes) act to define the developmental fate of each segment (Lewis 1978, Lewis 1982, comprehensively reviewed in Hughes and Kaufman 2002a). Mis-expression and loss of function of these genes often result in a homeotic transformation of a segment from one fate into the fate of another segment (e.g., Pultz et al. 1988; Gibson and Gehring 1988; Aplin and Kaufman 1997). Other hallmarks of Hox genes are their appearance in one (or several) cluster(s) and the fact that they usually obey temporal and spatial collinearity (reviewed in Gaunt 2018). The latter means that their onset of expression and expression along the AP axis correlates with their position in the clusters. A Hox gene that is located more upstream in a cluster usually starts to be expressed earlier (temporal aspect), and its anterior border of expression is located more anteriorly in the body (spatial aspect) than that of a neighboring Hox gene.

The ancestral arthropod Hox cluster contains ten genes (Grenier et al. 1997; Cook et al. 2001), but in higher insects like *Drosophila*, two of these genes, *Hox3* (i.e., *bicoid* (*bcd*) and *zerknüllt* (*zen*)) and *fushi-tarazu* (*ftz*), have lost their homeotic function and instead acquired new expression patterns and functions during development (reviewed in Akam et al. 1994, Damen 2002, Hughes and Kaufman 2002a, Pick 2016). In *Drosophila*, the Hox genes are present in the form of two clusters, the Antennapedia complex containing the genes *labial* (*lab*), *proboscipedia* (*pb*), *Hox3*/*zen*/*bcd*, *Deformed* (*Dfd*), *Sex combs reduced* (*Scr*), *fushi-tarazu* (*ftz*), and *Antennapedia* (*Antp*) and the Bithorax complex containing *Ultrabithorax* (*Ubx*), *abdominal-A* (*abdA*), and *Abdominal-B* (*AbdB*). The Hox genes of the Antennapedia complex are predominantly expressed in the anterior body regions of the developing fly embryo and the genes of the Bithorax complex more posteriorly (reviewed in Hughes and Kaufman 2002a).

The function of the Hox genes appears to be highly conserved in arthropods as evident from loss-of-function experiments and comparable gene expression patterns in a constantly increasing number of investigated species that represent all main groups of arthropods (e.g., Damen et al. 1998; Telford and Thomas 1998; Hughes and Kaufman 2000; Hughes and Kaufman 2002b; Deutsch and Mouchel-Vielh 2003; Janssen and Damen 2006; Schwager et al. 2007; Pavlopoulos et al. 2009; Sharma et al. 2012; Serano et al. 2016; Gainett et al. 2021). Beyond that, gene expression data also suggest that the function of Hox genes is conserved in the closest relatives of Arthropoda, the water bears (Tardigrada) and the velvet worms (Onychophora) (Eriksson et al. 2010; Janssen et al. 2014; Smith et al. 2016). The Hox genes therefore represent key conserved developmental factors in AP body patterning and the diversification of the arthropod body plan. Differences in the segmental fate and thus morphology including the set of appendages such segments may be equipped with are often correlated directly or indirectly with the function of these homeotic selector genes. The posterior Hox genes *Antp*, *Ubx*, *abdA*, and *AbdB*, for example, acquired repressive and/or modifying functions on appendage development and identity in arthropods including chelicerates (Levine et al. 1983; Carroll et al. 1986; Mahfooz et al. 2007; Liubicich et al. 2009; Robertson and Mahaffey 2009; Pavlopoulos et al. 2009; Hsia et al. 2010; Xiang et al. 2011; Khadjeh et al. 2012; Konopova and Akam 2014; Refki et al. 2014; Martin et al. 2016). For two groups of chelicerates, it has recently been shown that the pattern of the posterior Hox genes is largely overlapping in the homonomous opisthosoma of a harvestman (Opiliones) but staggered in the heteronomous opisthosoma of a scorpion (Scorpiones) (Sharma et al. 2012; Sharma et al. 2014a).

Spiders (Araneae) represent another group of chelicerate arthropods that have become important research organisms for evolutionary and developmental research (EvoDevo) during the last few decades. Most research in this field has been performed on true spiders belonging to the subgroup Entelegynae such as the cobweb spider *Parasteatoda tepidariorum* (e.g., Hilbrant et al. 2012; Oda and Akiyama-Oda 2020) and the American wandering spider *Cupiennius salei* (e.g., McGregor et al. 2008). In comparison, comparable research on their sister group the Haplogynae (or Synspermiata (i.e., Haplogynae excluding Filistatidae and Leptonetidae (Garrison et al. 2016)) is rather rare (e.g., Turetzek and Prpic 2016; Königsmann et al. 2017) and so are the data from the more distantly related Mygalomorphae (e.g., tarantulas) that form the sister group to Entelegyne and Haplogynae (Entelegyne+Haplogynae = “true spiders” (Araneomorphae)) (e.g., Pechmann and Prpic 2009; Pechmann 2020). Morphologically, Entele- and Haplogynae are very alike, and the main difference between them is represented by the female mating apparatus (Garrison et al. 2016). Mygalomorphae, however, display more morphological differences. The first obvious difference is the position of the chelicerae, which is orthognath in tarantulas but labidognath in true spiders (e.g., Foelix 2010). Other morphological differences between true spiders and tarantulas concern their opisthosoma. The opisthosomal appendages of spiders are either lost or have been heavily modified to fulfill new functions, often representing evolutionary novelties. In both true spiders and tarantulas, the second opisthosomal segment (O2) carries a pair of book lungs, which represent complex breathing organs. In tarantulas, this is even the case on the third opisthosomal segment (O3), but in true spiders, the book lungs on O3 evolved into a simpler tracheal tube for gas exchange (e.g., Foelix 2010; Sharma 2017). On O4 and O5, true spiders carry sets of spinnerets, an evolutionary novelty unique to spiders among the chelicerates. In tarantulas, however, the anterior spinnerets (on O4) only develop rudimentarily and disappear later during embryonic development (Pechmann and Prpic 2009, Pechmann 2020, reviewed in Mariano-Martins et al. 2020). Since Mesothelae, the most basally branching group of spiders, possess well-developed spinnerets on both O4 and O5, the situation in the tarantula is likely derived. Indeed, spontaneous reactivation of *Distal-less* (*Dll*) expression and development of spinnerets on O4 in the tarantula *Tliltocatl albopilosum* have been observed suggesting that the genetic downregulation of *Dll* and thus spinneret development is not very stable, possibly depending on a single or few genetic changes (Pechmann 2020). In *Drosophila* for example, Ubx and abdA repress *Dll* expression and thus appendage development in the abdomen providing a relatively “simple” regulatory mechanism (e.g., Vachon et al. 1992).

In this paper, we begin to investigate the genetics that control the differences in morphology of the opisthosoma between true spiders and tarantulas. Therefore, we studied the expression of Hox genes that are expressed in the opisthosoma of spiders, the genes of the Bithorax complex (*Ubx*, *abdA*, and *AbdB*), and the most posterior gene of the Antennapedia complex (*Antp*). These genes are all present in the form of two copies in spiders, which is the result of a whole genome duplication (WGD) in the last common ancestor of Arachnopulmonata (i.e., Scorpiones+Tetrapulmonata (Sharma et al. 2014b)) (e.g., Schwager et al. 2007; Schwager et al. 2017; Harper et al. 2021). We compared the expression data of the tarantula with previously published data on Hox gene expression and function in true spiders (recently reviewed in Turetzek et al. 2022). Our data show that most of the posterior Hox genes are expressed in identical or very similar patterns in all investigated spiders. Expression of *abdA* genes, however, differs in tarantulas and true spiders, especially with respect to the opisthosomal limb buds. We therefore suggest that *abdA* may be involved in the evolution of some of the opisthosomal differences in spiders.

## Methods

### Phylogenetic analysis

Reciprocal BLAST searches (tBLASTn) were performed against the published embryonic transcriptomes of *Acanthoscurria geniculata* (Pechmann 2020), *Pholcus phalangioides* (Janssen et al. 2015), and *Cupiennius salei* (Samadi et al. 2015) using protein sequences of Hox genes from the true spider *Parasteatoda tepidariorum* (Harper et al. 2021) as queries. Protein sequences of the potential tarantula Hox genes were aligned with T-Coffee using default parameters in MacVector version 12.6.0 (Supplementary Files 1 and 2). A subsequent phylogenetic analysis was performed with MrBayes (Huelsenbeck and Ronquist 2001) as previously described in Panara et al. (2019), applying 0.5 million cycles for the Metropolis-coupled Markov chain Monte Carlo (MCMCMC) analysis. Unique sequence identifiers of all genes are summarized in Supplementary File 3.

### Gene cloning

Genes were amplified by means of RT-PCR using gene-specific primers (Supplementary File 3) and cDNA reverse transcribed from total RNA of a mix of embryonic stages. For all gene fragments, additionally, a nested PCR was performed to boost the PCR results and to apply a higher degree of specificity; for the nested PCR, the first PCR served as a template. We identified two non-overlapping fragments of *A. geniculata Antp_B* and *abdA_B* respectively in an embryonic transcriptome (Supplementary File 3). Primers for the amplification of these genes were placed in the N-terminal fragment (forward primers) and the C-terminal fragment (backward primers). The complete sequences amplified with these primers, bridging the two non-overlapping fragments of *A. geniculata Antp_B* and *abdA_B*, have been deposited in Supplementary File 3. All gene fragments have been ligated into a pCR-II vector (TA Cloning Kit Dual Promoter, Invitrogen) and sequenced from both directions by a commercially offered sequencing service (Macrogen).

### Animal husbandry and in situ hybridization

Embryos of the tarantulas *A. geniculata* and *T. albopilosum* (earlier synonym *Brachypelma albopilosum* (Mendoza and Francke 2020)) were obtained and treated as described in Pechmann and Prpic (2009) and Pechmann (2020). Embryos of *C. salei* and *P. phalangioides* were treated as described in Prpic et al. (2008). In situ hybridizations were performed as previously described by Pechmann (2020) and Janssen et al. (2018). The developmental staging of *A. geniculata* and *T. albopilosum* embryos follows Pechmann (2020). Staging of *P. phalangioides* and *C. salei* follows Turetzek and Prpic (2016) and Wolff and Hilbrant (2011), respectively.

### Data documentation

A MZ-FLIII Leica dissection microscope equipped with a Leica DC490 digital camera and an external UV-light source was used to photograph stained embryos. Whenever necessary and justified, linear adjustments were performed on color, contrast, and brightness using the image-processing software Adobe Photoshop CC 2018.

## Results

### Tarantula Hox genes

**Fig. 1:**
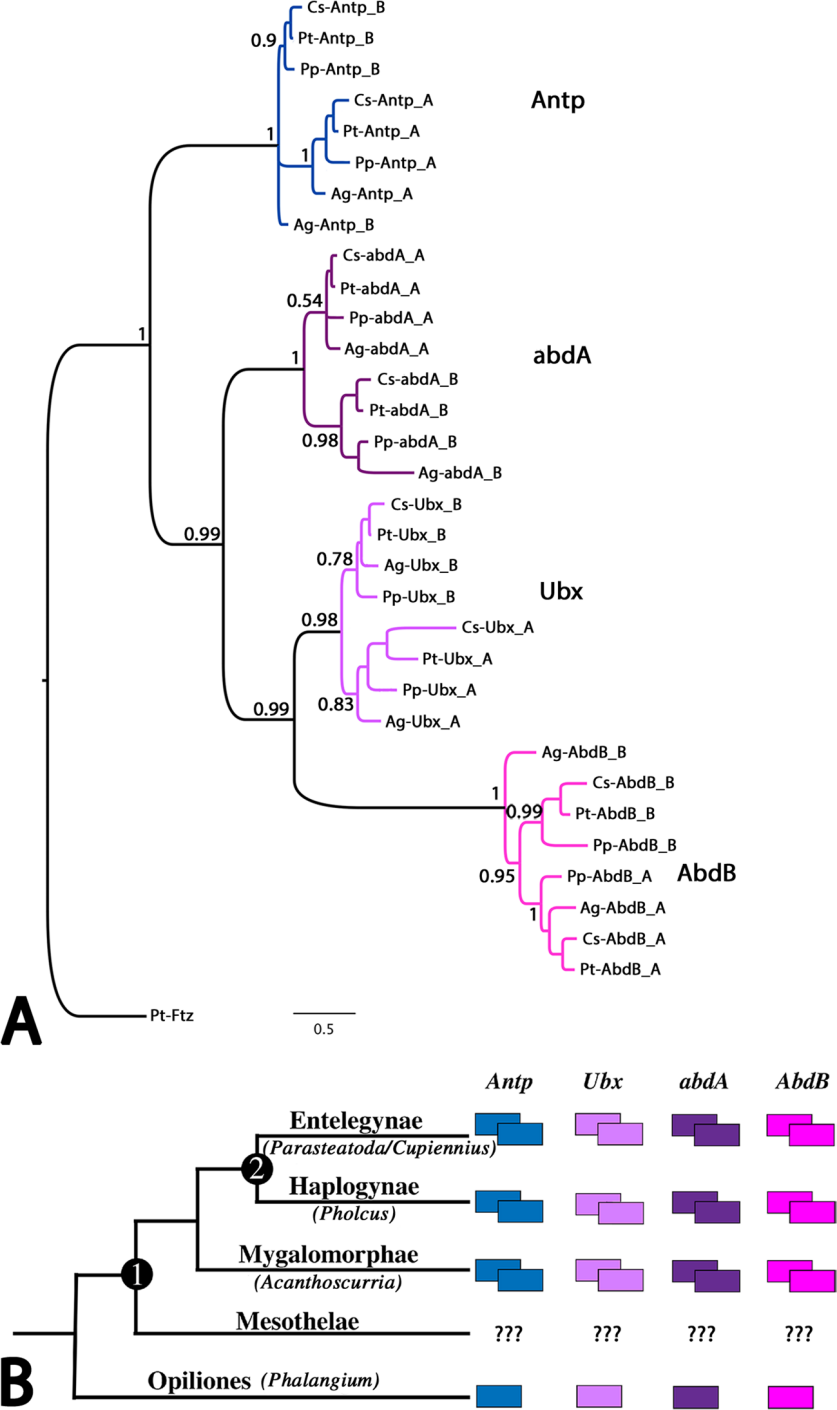
Phylogeny and Hox gene complement of spiders. **A** Phylogenetic tree of all posterior Hox genes from all selected spider species; the tree is based on the complete sequences of the conceptually translated proteins. The sequence of *P. tepidariorum* fushi-tarazu (Ftz) serves as an outgroup. Colors represent the Hox gene sub-families. Species abbreviations are as follows: Ag, *Acanthoscurria geniculata*; Cs, *Cupiennius salei*; Pp, *Pholcus phalangioides*; Pt, *Parasteatoda tepidariorum*. Numbers at the tree edges represent posterior probability values. The scale bar represents 0.5 amino acid substitutions per site. Nomenclature of the Hox genes follows Schwager et al. (2017). Note that *Cs-Antp_A* was previously described as *Cs-Antp*, *Cs-abdA_B* was described as *Cs-abdA1*, *Cs-abdA_A* was described as *Cs-abdA2*, *Cs-Ubx_A* was described as *Cs-Ubx2*, and *Cs-Ubx_B* was described as *Cs-Ubx1* (Damen et al. 1998). **B** Schematic cladogram of posterior Hox genes in the investigated spiders and the harvestman (Opiliones) *Phalangium opilio* (Sharma et al. 2012). Note that all posterior Hox genes are duplicated in the investigated arachnopulmonate species (denoted by an encircled “1”). The encircled “2” marks the node representing true spiders (Entelegynae+Haplogynae). Note that no data exist about the Hox gene content in “segmented spiders” (Mesothelae)

We identified two copies (paralogs) of each of the posterior Hox genes, *Antp*, *Ubx*, *abdA*, and *AbdB* (labeled with the postfix “A” and “B” respectively, following the nomenclature of Schwager et al. 2017) in the tarantula *A. geniculata* (Fig. 1A, B). In our phylogenetic analysis, each of the identified Hox genes clusters with total support (posterior probability of 1) with representatives of their class of Hox genes from the true spider *P. tepidariorum* (Fig. 1A) (see Harper et al. (2021) for a more comprehensive analysis of chelicerate Hox genes, but note that they only identified one copy of *AbdB* in *A. geniculata*).

### Expression of tarantula *Antennapedia* genes

**Fig. 2:**
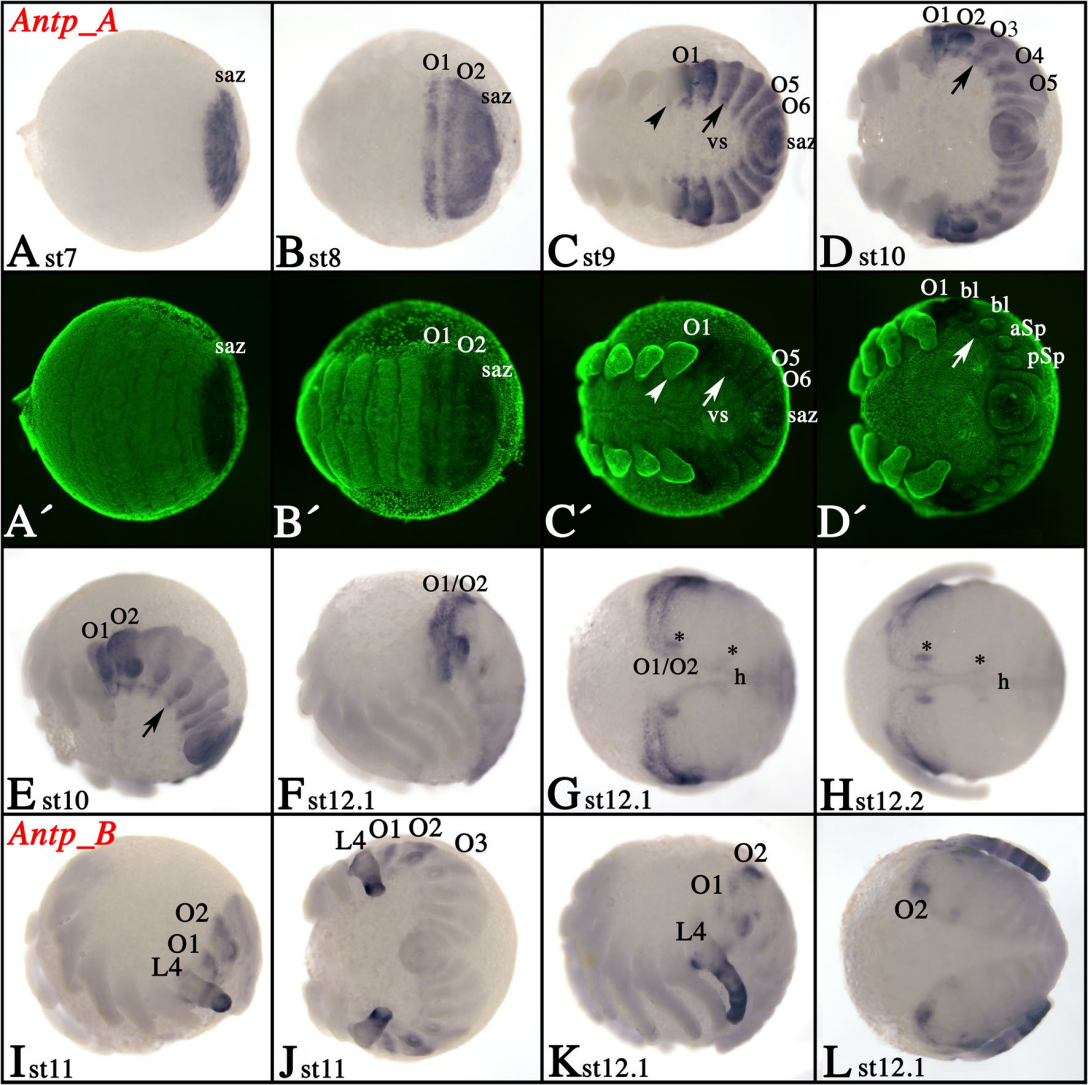
Expression of tarantula *Antp* genes. **A**–**H** Expression of *Antp_A*; **I**–**L** expression of *Antp_B*. In all panels, anterior is to the left. Panels **E**, **F**, **I**, and **K** present lateral views. Panels **G**, **H**, and **L** present dorsal views. **A′**–**D′** SYBR green counter-stained embryos of the embryos shown in **A**–**D**. Developmental stages are indicated. The arrowhead in panel **C** points to most anterior dot-like expression in the ventral nervous system (VNS). Arrows point to dot-like expression in the VNS of more posterior segments. Asterisks in panels **G** and **H** mark expression in the heart. Abbreviations: aSp, anterior spinneret; bl, book lung; h, heart; L, leg-bearing segment; O, opisthosomal segment; pSp, posterior spinneret; saz, segment addition zone; vs, ventral sulcus

Expression of *Antp_A* starts around stage 7 in the form of a broad domain that covers the complete segment addition zone (SAZ) (Fig. 2A). When the opisthosomal (O) segments begin to bud off from the SAZ, *Antp_A* is expressed in these newly formed segments (Fig. 2B). In subsequent stages, as more posterior segments are added, *Antp_A* is consistently expressed in these segments (Fig. 2C, D). Expression in O1 and O2 is stronger than in more posterior segments (Fig. 2C, D). Ventral to the base of the opisthosomal limb buds, *Antp_A* is expressed in dot-like domains in the developing ventral nervous system (VNS) (arrows in Fig. 2C–E). A single dot of expression that may also be correlated with the VNS is in the fourth walking leg-bearing segment (L4), ventral to the base of this leg (arrowhead in Fig. 2C). Expression is also in the ventral sulcus (the thin layer of ectodermal cells between the splitting halves of the germ band proper) (Fig. 2C). This expression is relatively weak (or appears weak because the layer of cells is very thin). A stronger domain of expression in the ventral sulcus is seen at late stages in a transverse stripe demarcating the most anterior expansion of *Antp_A* expression (arrow in Supplementary File 4A). Similarly, the dorsal field (DF) (see Prpic and Pechmann (2022) for further information on this tissue) expresses *Antp_A* (asterisk in Supplementary File 4B). At late developmental stages, expression in segments posterior to O2 begins to fade; expression in O1 and O2, however, remains strong (Fig. 2F, G). Expression posterior to O2 is now mainly restricted to the developing dorsal tube (=heart) and associated structures (cf. Janssen and Damen 2008 for information on spider heart formation) (asterisks in Fig. 2G, H).

Expression of *Antp_B* appears much later than that of *Antp_A* and expands anteriorly into L4 (Fig. 2I–L and Supplementary File 4C (inlay)). In the leg of L4, *Antp_B* is expressed in several rings (Fig. 2I–L and Supplementary File 4C (inlay)). Like *Antp_A*, *Antp_B* is expressed most strongly in O1 and O2 (Fig. 2I–L). Expression in O3 and more posteriorly located segments is very weak, almost below the detectable level (Fig. 2J). Later during development, *Antp_B* is also expressed in the developing heart (Fig. 2L).

### Expression of tarantula *Ultrabithorax* genes

**Fig. 3:**
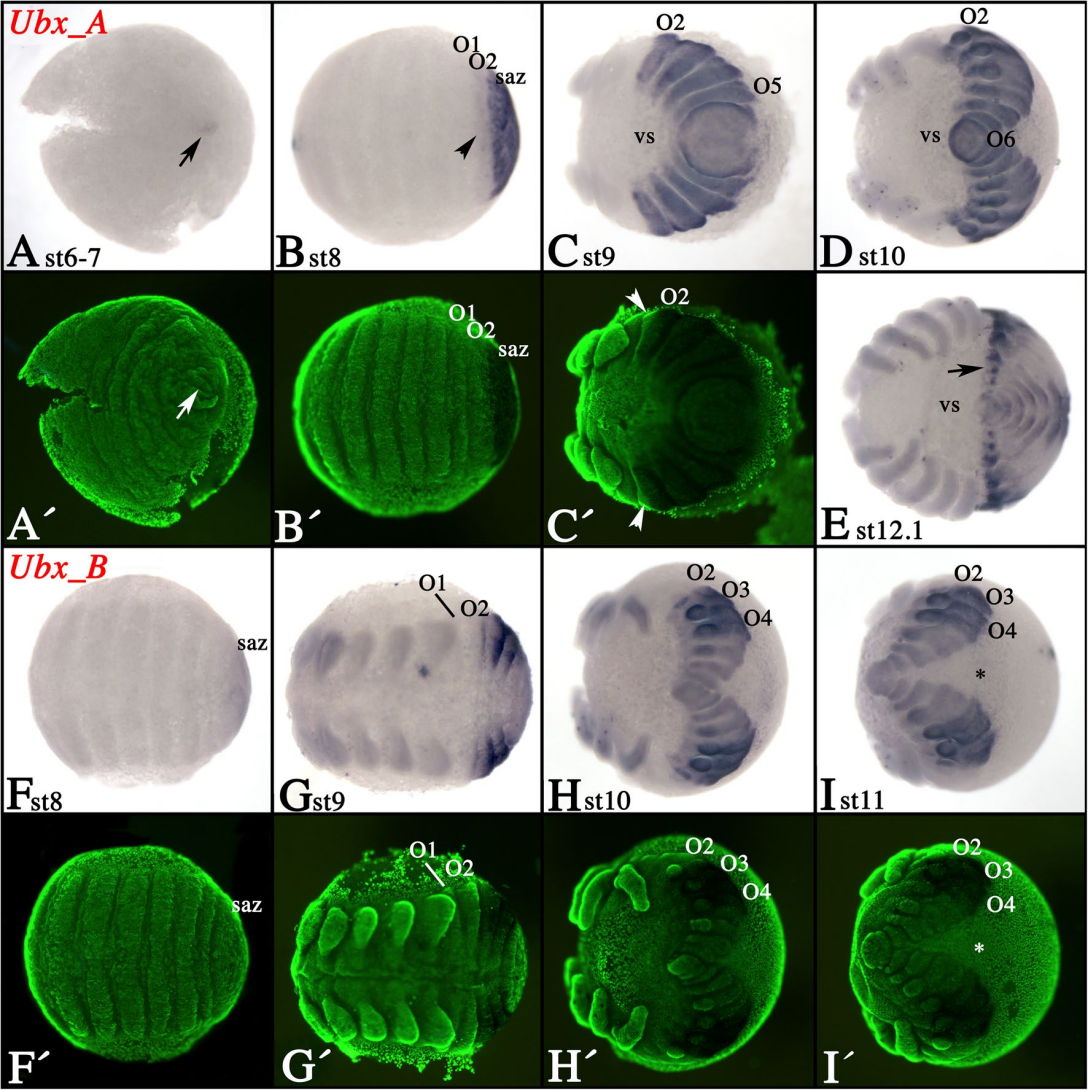
Expression of tarantula *Ubx* genes. **A**–**E** Expression of *Ubx_A*; **F**–**I** expression of *Ubx_B*. In all panels, anterior is to the left. All panels present ventral views except panel **I** that shows view on posterior of the embryo. **A′**–**C′**, **F′**–**I′** SYBR green counter-stained embryos of the embryos shown in **A**–**C** and **F**–**I**. Developmental stages are indicated. The arrow in panel **A** points to expression in the center of the SAZ. The arrowhead in panel **B** points to weaker expression in O2. Arrowheads in panel **C′** mark expression restricted to the posterior region of O2. The asterisk in panel **I** marks expression in the dorsal field. Abbreviations as in Fig. 2

*Ubx_A* is first expressed at stage 6–7 in the form of a small dot in the center of the SAZ (arrow in Fig. 3A). This remains the only expression until the O2 segment buds off the SAZ. At this point, *Ubx_A* is weakly expressed in O2 (arrowhead in Fig. 3B). In the following stages, O2 and all segments posterior to O2 express *Ubx_A* strongly (Fig. 3C–E); note that the anterior border of expression lies approximately in the middle of O2 (arrowheads in Fig. 3C′). Like *Antp_A*, *Ubx_A* is also expressed in the ventral sulcus and the DF ectoderm (Fig. 3C–E). Later during development, expression appears in the form of a dot-like pattern of stronger expression in the developing VNS (arrow in Fig. 3E), and dorsal expression is mainly restricted to the developing heart and associated tissue (Supplementary File 4D, E).

Expression of *Ubx_B* starts slightly later than that of *Ubx_A* (Fig. 3F). The most anterior extension of *Ubx_B* is posterior in O2 and thus slightly more posterior than that of *Ubx_A* (Fig. 3G–I). Expression in O3 and O4 is stronger than in more posterior segments (Fig. 3H, I). Later during development, during and after dorsal closure, expression of *Ubx_B* is expressed in all dorsal tissue and not like *Ubx_A* restricted to the heart (Supplementary File 4F-H). Expression is also in the tissue of the DF that is associated with the *Ubx_B*-expressing segments (Fig. 3H, I; asterisk in panel I).

### Expression of tarantula *abdominal-A* genes

**Fig. 4:**
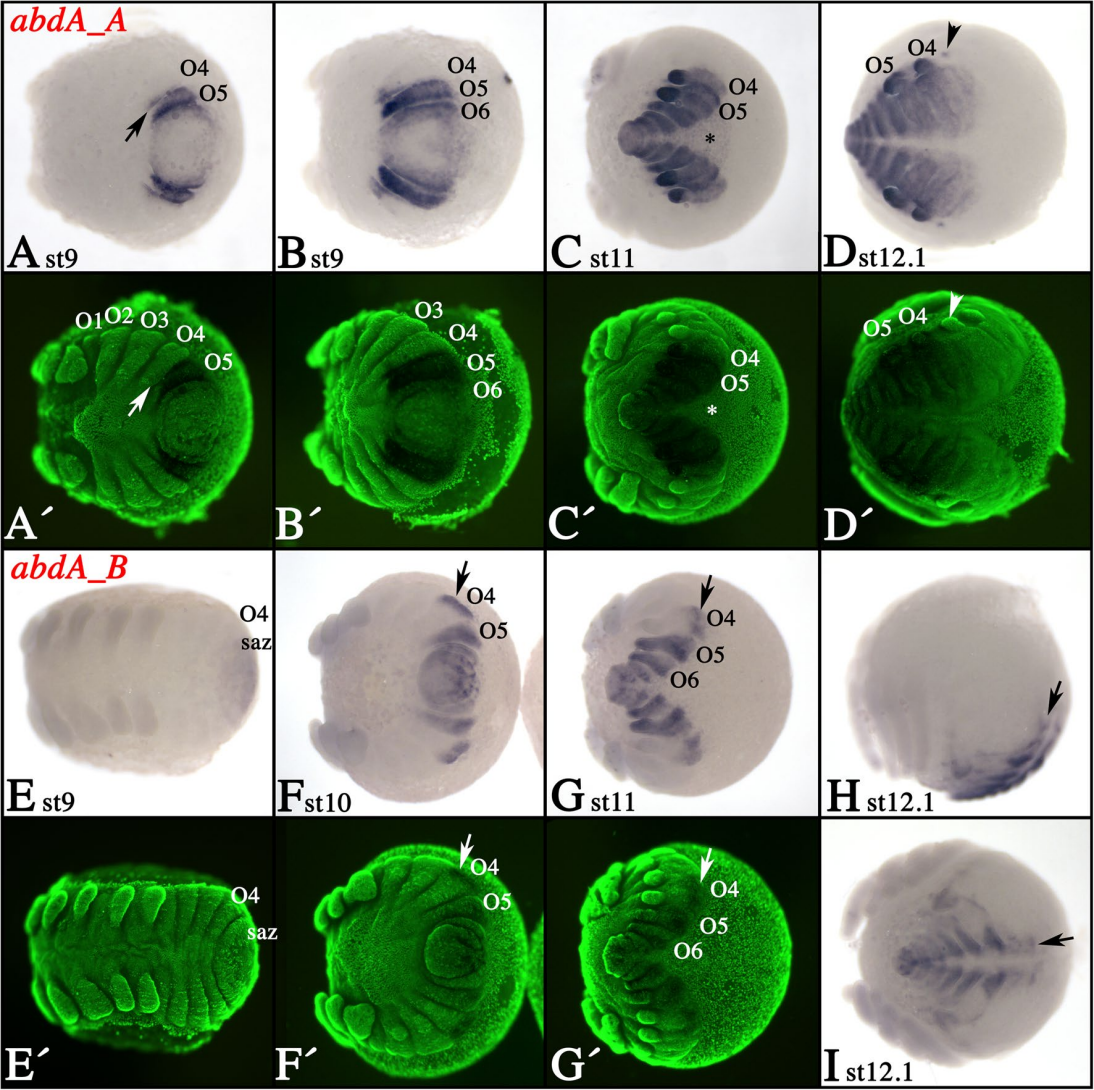
Expression of tarantula *abdA* genes. **A**–**D** Expression of *abdA_A*; **E**–**I** expression of *abdA_B*. In all panels, anterior is to the left. All panels present views of the posterior of the embryo, except panels **A** and **E** that present ventral views and panel **H** which is a lateral view. **A′**–**G′** SYBR green counter-stained embryos of the embryos shown in **A**–**G**. Developmental stages are indicated. The arrow in panel **A** marks expression in the posterior of O4. The asterisk in panel **C** marks expression in the dorsal field. The arrowhead in panel **D** points to dot-like expression in O3. Arrows in panels **F**–**I** mark dorsal expression of *abdA_B* in O4; note that there is no ventral expression, including the limb buds. Abbreviations as in Fig. 2

Expression of *abdA_A* appears at stage 9 in the form of a ring around the SAZ and in the posterior of the newly forming O4 segment (arrow in Fig. 4A). Shortly thereafter, expression in O4 is restricted to ventral tissue, while the newly formed O5 expresses *abdA_A* throughout the segment (Fig. 4B). Later, the whole of O4 also expresses *abdA_A*, as do all newly forming posterior segments (Fig. 4C, D). A dot of expression is visible ventral to the base of the limb bud in O3 (arrowhead in Fig. 4D and Supplementary File 4I); note that similar dots are also present ventral to the limb buds in more posterior segments (Fig. 4C). This expression is likely associated with the developing VNS. In addition, *abdA_A* is expressed in the DF (asterisk in Fig. 4C).

Expression of *abdA_B* also appears around stage 9 when it is restricted to the SAZ (Fig. 4E). When O4 buds off the SAZ, *abdA_B* is expressed in its dorsal portion (arrows in Fig. 4F–I). The ventral region of this segment including the limb buds of the rudimentary anterior spinnerets is never seen to express *abdA_B*. In O5 and more posterior segments, *abdA_B* was detected in all tissues (Fig. 4G). Later during development, during dorsal closure, expression of *abdA_B* is mainly restricted to the developing heart and associated structures like the alary muscles (cf. Janssen and Damen 2008) (Fig. 4H, I).

### Expression of *abdA* genes in other spiders

We investigated the expression of *abdA* genes in other spiders in order to compare the expression between tarantulas and true spiders. Expression of both *abdA* paralogs has been described in detail for *P. tepidariorum* (Schwager et al. 2017). For *C. salei*, however, only one paralog, *abdA_B*, has been described previously (Damen et al. 1998). The second paralog, *abdA_A*, is expressed in the fourth opisthosomal segment (O4) including its limb buds and all tissue posterior to O4 (Supplementary File 5A, B). In *P. phalangioides*, *abdA_A* and *abdA_B* are expressed in the same conserved pattern as shown for *P. tepidariorum* and *C. salei* including the limb buds in O4 and O5 (Supplementary File 5C-H). In all of the investigated true spiders, both *abdA* genes are thus expressed in the developing spinnerets (O4 and O5) throughout development.

**Fig. 5:**
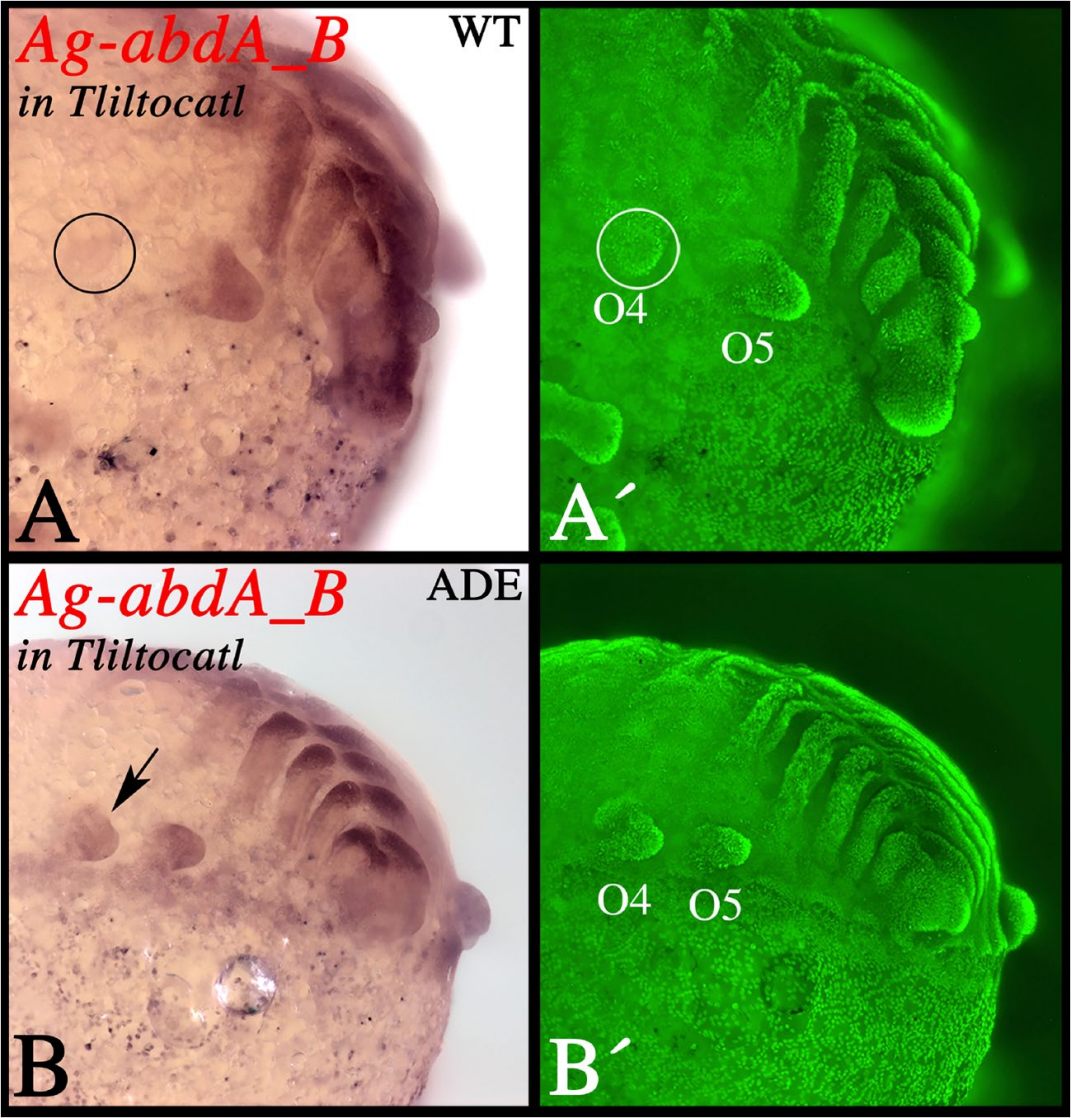
Expression of *abdA_B* in abnormally developing embryos (ADEs) of *T. albopilosum*. **A** A magnification of the posterior part of a wild type (WT) embryo, expression of *abdA_B*. Note the lack of expression in the rudimentary anterior spinneret in O4 (encircled). **B** Expression of *abdA_B* in an ADE that develops anterior spinnerets in O4 (arrows). Note that these spinnerets express *abdA_B*. **A′**, **B′** SYBR green counter-staining of the embryos shown in panels **A** and **B**. Abbreviation: O, opisthosomal segment

The anti-sense probes of *A. geniculata abdA_A* and *abdA_B* detect transcripts in another mygalomorph species, the curly hair tarantula *T. albopilosum*. Expression of both genes is identical in both species including the lack of expression of *abdA_B* in the O4 limb buds (Supplementary File 6). Interestingly, in some abnormally developing embryos (ADEs) of *T. albopilosum*, which show the ectopic development of the anterior pair of spinnerets on O4, also the expression of *abdA_B* is activated in these ectopic limb buds (Fig. 5, cf. panels A and B) (discussed below).

### Expression of tarantula *Abdominal-B* genes

**Fig. 6:**
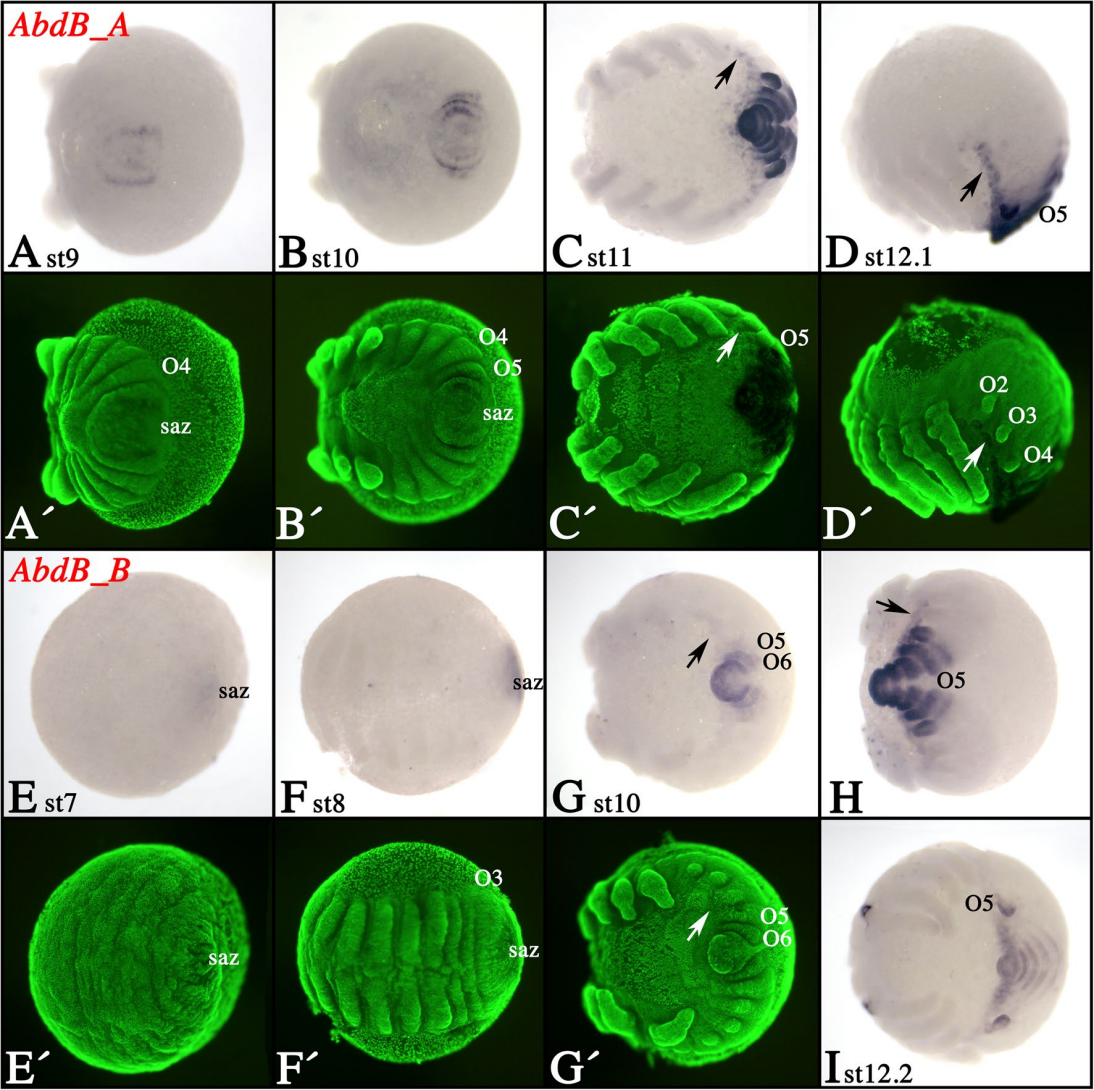
Expression of tarantula *AbdB* genes. **A**–**D** Expression of *AbdB_A*; **E**–**I** expression of *AbdB_B*. In all panels, anterior is to the left. All panels present ventral views, except panel **A** and **H** (posterior view) and panel **D** (lateral view). **A′**–**G′** SYBR green counter-stained embryos of the embryos shown in **A**–**G**. Developmental stages are indicated. Arrows in panels **C**, **D**, **G**, and **H** mark anteriorly extended expression (compared to the main domain of segmental Hox gene expression). Abbreviations as in Fig. 2

*AbdB_A* is first expressed around stage 9 in the form of a solid domain in the center of the SAZ and a circle in the periphery of the SAZ from which the O5 segment will soon after bud off (Fig. 6A). When O5 forms, *AbdB_A* is expressed in the complete segment (Fig. 6B, C). Likewise, *AbdB_A* is expressed in all more posterior segments (Fig. 6C, D). Ventrally, in the VNS, expression of *AbdB_A* extends anteriorly into O1 (arrows in Fig. 6C, D).

Expression of *AbdB_B* in the SAZ starts at stage 7 (Fig. 6E, F). When O5 forms, it expresses *AbdB_B*. At later developmental stages, *AbdB_B* is expressed in O5 and all more posteriorly located segments (Fig. 6H, I). In the VNS, expression of *AbdB_B* extends into O3 (arrows in Fig. 6G, H).

### Expression of Abdominal-B genes in the haplogyne spider Pholcus phalangioides

*P. phalangioides AbdB_A* is expressed in the developing ventral nervous system of O3 and more posterior opisthosomal segments; a small dot of expression is in the ventral tissue of O2 (Supplementary File 7A, B). The posterior spinnerets in O5 express *AbdB_A*, but in the anterior spinnerets, expression is restricted to their posterior half (Supplementary File 7A, B).

Expression of *P. phalangioides AbdB_B* is first restricted to the segment addition zone of the early embryo (Supplementary File 7C). Note that the expression of *AbdB_B* starts much earlier than that of *AbdB_A* and that the former clearly disobeys the collinearity rule. Expression of *AbdB_B* does not extend as much toward the anterior in the ventral nervous system as that of *AbdB_A* (Supplementary File 7D-F). At late developmental stages, expression of *AbdB_B* is also in the developing book lungs in O2 (Supplementary File 7E, F)

## Discussion

### Widely conserved expression of *Antp*, *Ubx*, and *AbdB* in the opisthosoma of spiders

In true spiders and the tarantula *A. geniculata*, *Antp_A* is expressed in all opisthosomal segments including all opisthosomal limb buds (Fig. 2) (Damen et al. 1998; Khadjeh et al. 2012; Schwager et al. 2017). The only difference concerns expression in the most posterior prosomal segment that carries the fourth leg pair (L4). While in *A. geniculata* the legs of L4 do not express *Antp_A*, *Antp_A* is clearly expressed in this pair of appendages in true spiders (Fig. 2) (Damen et al. 1998, Khadjeh et al. 2012, Schwager et al. 2017). The function of Antp_A in the limb buds of O4 is unclear, but it could represent an ancestral feature of at least arachnopulmonate chelicerates as this pattern is also present in a scorpion (Sharma et al. 2014a). Expression of *Antp_B* is also very similar in *P. tepidariorum* and *A. geniculata* (data on *Antp_B* expression from other previously studied spiders such as *C. salei* are not available). Both genes extend into L4, are strongly expressed in O1 to O3, and are weakly (or not at all) expressed in more posterior opisthosomal segments. The only apparent difference is that *Antp_B* is expressed in the form of a single dot in each of the spinnerets in *P. tepidariorum*, but not in *A. geniculata* (Fig. 2) (Schwager et al. 2017). Since comparative data on the expression of *Antp_B* in spiders other than these two species is lacking, it remains unclear which state is ancestral. However, since expression in *P. tepidariorum* is in both spinnerets, but in neither spinneret (neither the rudimentary anterior (O4) nor the well-developed posterior (O5)) in *A. geniculata*, this expression does not contribute to the different morphology of the opisthosoma of spiders and tarantulas.

The most anterior domain of *Ubx* expression lies in the O2 segment of spiders. In *A. geniculata*, *P. tepidariorum*, and *C. salei*, this anterior domain is conserved for *Ubx_A* and *Ubx_B* respectively. *Ubx_A* is expressed in most of O2 except for its vey anterior region, and the anterior border of *Ubx_B* is slightly more posterior than that of *Ubx_A* (Fig. 3) (Damen et al. 1998; Schwager et al. 2007; Schwager et al. 2017). Expression of one *Ubx* gene, *Ubx_B*, has also been investigated in the haplogyne spider *P. phalangioides* where it is expressed in a comparable pattern as in the entelegyne spiders (Turetzek 2011). Interestingly, stronger expression of *Ubx_B* is present in O3 in all investigated spiders (Fig. 3) (Damen et al. 1998; Schwager et al. 2017; Turetzek 2011). In the tarantula, however, expression of *Ubx_B* is also enhanced in O4 (Fig. 3). The function of this enhanced expression is unclear, but it appears not to be correlated with the formation of different opisthosomal appendages because these segments bear different appendages, tracheae (O3) in true spiders and book lungs (O3) and rudimentary spinnerets (O4) in *A. geniculata*. Hence, there is no direct correlation of enhanced expression and a specific type of appendage.

In all previously investigated spiders, *AbdB_A* expression appears around stage 9 in O5 and is then also expressed in all more posterior segments (Fig. 6 and Supplementary File 7) (Turetzek 2011; Schwager et al. 2017). At later developmental stages, expression extends into the posterior half of the O4 segment in true spiders (but not the tarantula), including the posterior of the developing limb bud in O4 (Fig. 6 and arrowheads in Supplementary File 7) (Schwager et al. 2017). This difference could be correlated with the rudimentary state of the anterior spinnerets in mygalomorph spiders. Expression of *AbdB_B* starts very early during spider germ band formation and thus clearly disobeys the temporal collinearity rule (Fig. 6E, F and Supplementary File 7C) (Damen and Tautz 1999; Schwager et al. 2017). The complete O5 expresses *AbdB_B*, but expression in the developing nervous system is also seen in O3 and O4 (except for *P. phalangioides* where expression of *AbdB_B* does not extend toward the anterior into the VNS (Supplementary File 7E, F)). In contrast to *P. tepidariorum*, *P. phalangioides AbdB_B* is also expressed in the posterior region of the developing anterior spinnerets (Supplementary File 7E, F). In all spiders, except the tarantula, *AbdB_B* is expressed at the base of the limb bud of the second opisthosomal segment (arrows in Supplementary File 7E, F). This expression is likely associated with the development of the genitalia (Damen and Tautz 1999), a function of *AbdB* genes that appears to be also conserved in other chelicerates (Sharma et al. 2014a) and arthropods in general (e.g., Sánchez-Herrero et al. 1985; DeLorenzi and Bienz 1990; Kelsh et al. 1993; Averof and Akam 1995; Copf et al. 2003; Brena et al. 2005). It was therefore surprising to find that neither of the two *AbdB* genes is expressed in the developing genital opening or the region where this structure is supposed to develop in the tarantula *A. geniculata* (Fig. 6G–I). It is either that *A. geniculata* represents an exception from the rule, and *AbdB* genes indeed are not involved in the development of the genitalia, or that expression of *AbdB_B* and development of the associated structures are delayed in tarantulas compared to other spiders. In scorpions, for example, the genital opening forms later during development, and thus, there is no expression of *AbdB* in this region of the developing embryo (Sharma et al. 2014a). Possibly, such delayed development of the genitalia could be correlated with the slow development of tarantulas that usually need several years until they reach sexual maturity (e.g., Pechmann 2020).

It was recently suggested that the function of AbdB_B in O2 of spiders is not correlated with the development of the genitalia but the development of the book lungs (Turetzek et al. 2022). Our data, however, do not support this idea because despite the fact that tarantulas develop book lungs in both segments, O2 and O3, they do not express *AbdB_B*.

### *abdA* genes and their possible role in opisthosomal appendage development in spiders

In the true spiders, *P. tepidariorum*, *C. salei*, and *P. phalangioides*, *abdA* genes are strongly expressed in the developing spinnerets on O4 and O5 (Supplementary File 5) (Damen et al. 1998; Schwager et al. 2017). This pattern is also conserved for *abdA_A* in *A. geniculata* (Fig. 4C, D). Expression of *A. geniculata abdA_B*, however, is lacking from the ventral portion of the O4 segment, including the rudimentary anterior spinnerets (Fig. 4F–I). In another mygalomorph spider, the curly hair tarantula *T. albopilosum* (earlier synonym *Brachypelma albopilosum*), the *A. geniculata abdA_B* probe cross-hybridizes and detects the same pattern (Supplementary File 6). This represents the most striking difference in opisthosomal Hox gene expression in true spiders *versus* tarantulas and may be correlated with the repressed development of the anterior spinnerets in the latter. Although limb buds initially form on O4 in tarantulas, they disappear later during development, and consequently, no functional anterior spinnerets develop (e.g., Pechmann et al. 2010). Interestingly, spontaneously occurring development of anterior spinnerets in *T. albopilosum* is indeed linked with the expression of *Dll* in the anterior spinnerets (Pechmann 2020), which means that the rudimentary state of the anterior spinnerets is at least partially dependent on *Dll*.

In the fly *Drosophila*, AbdA inhibits appendage development on the abdomen by repressing *Dll* (Vachon et al. 1992; Castelli-Gair and Akam 1995), a gene that is also needed for appendage outgrowth in other arthropods including spiders (Cohen et al. 1989; Schoppmeier and Damen 2001; Sharma et al. 2013; Hiruta et al. 2018). In other arthropods, this repressive function of AbdA on limb development is likely conserved. In the crustacean *Artemia franciscana*, for example, *abdA* is expressed in the limb-bearing trunk region, but AbdA protein is not produced, suggesting that the presence of AbdA could repress limb development in this region (Hsia et al. 2010). In another crustacean, *Parhyale hawaiensis*, and a basally branching hexapod, the springtail *Orchesella cincta*, mutation of *abdA* inhibits limb diversification (Konopova and Akam 2014; Martin et al. 2016). In the pill millipede *Glomeris marginata*, a myriapod, posterior appendages form in the segments that express *abdA*, but the distal *Dll*-expressing region of the outgrowing limbs does not express *abdA*, suggesting that otherwise AbdA protein could repress *Dll* (Janssen and Damen 2006). It is thus likely that AbdA can repress or modify limb development in tarantulas as well and that this involves the repression of *Dll*.

Expression of *abdA* genes in the spinnerets of spiders is not surprising if its function is that of a repressor of leg development and/or that of a modifier of appendage development (from an ancestral locomotory type of appendage). But how could the absence of one paralog (*abdA_B*) repress spinneret development (as seen in O4)? One explanation could be that AbdA_B acts as an activator or maintenance factor of *Dll* expression and thus spinneret development in spiders, a function that would then be fundamentally different from the function of AbdA in *Drosophila*, where it acts as a repressor of *Dll*.

Since there are two paralogs of *abdA* in spiders (including tarantulas), one (in this case *AbdA_B*) could have evolved a new function, i.e., that of a “passive positive” factor of appendage development: If the AbdA_B protein of tarantulas lacks the repressor function that AbdA_A may possess, and if the affinity of AbdA_B to a shared AbdA-binding site of *Dll* (DCRE element) is higher than that of AbdA_A, then the latter cannot efficiently repress *Dll* expression (and thus limb growth/maintenance) in the presence of AbdA_B.

Additional support for this suggested theory comes from abnormally developing embryos (ADEs) of *A. geniculata*. Under certain circumstances (which remain unclear), embryos develop with elongated anterior spinnerets (O4) that resemble normally developing posterior spinnerets (O5). In a previous study, it has been shown that in these ADEs, *Dll* is expressed in the anterior spinnerets, although this is not the case in normally developing embryos (Pechmann 2020). If our theory holds true, one would expect ectopic expression of *abdA_B* in the anterior spinnerets of the *A. geniculata* ADEs, and indeed in ADEs representing the same batch of embryos that express *Dll* in the anterior spinnerets, *abdA_B* is prominently expressed in the anterior spinnerets (Supplementary File 6).

**Fig. 7:**
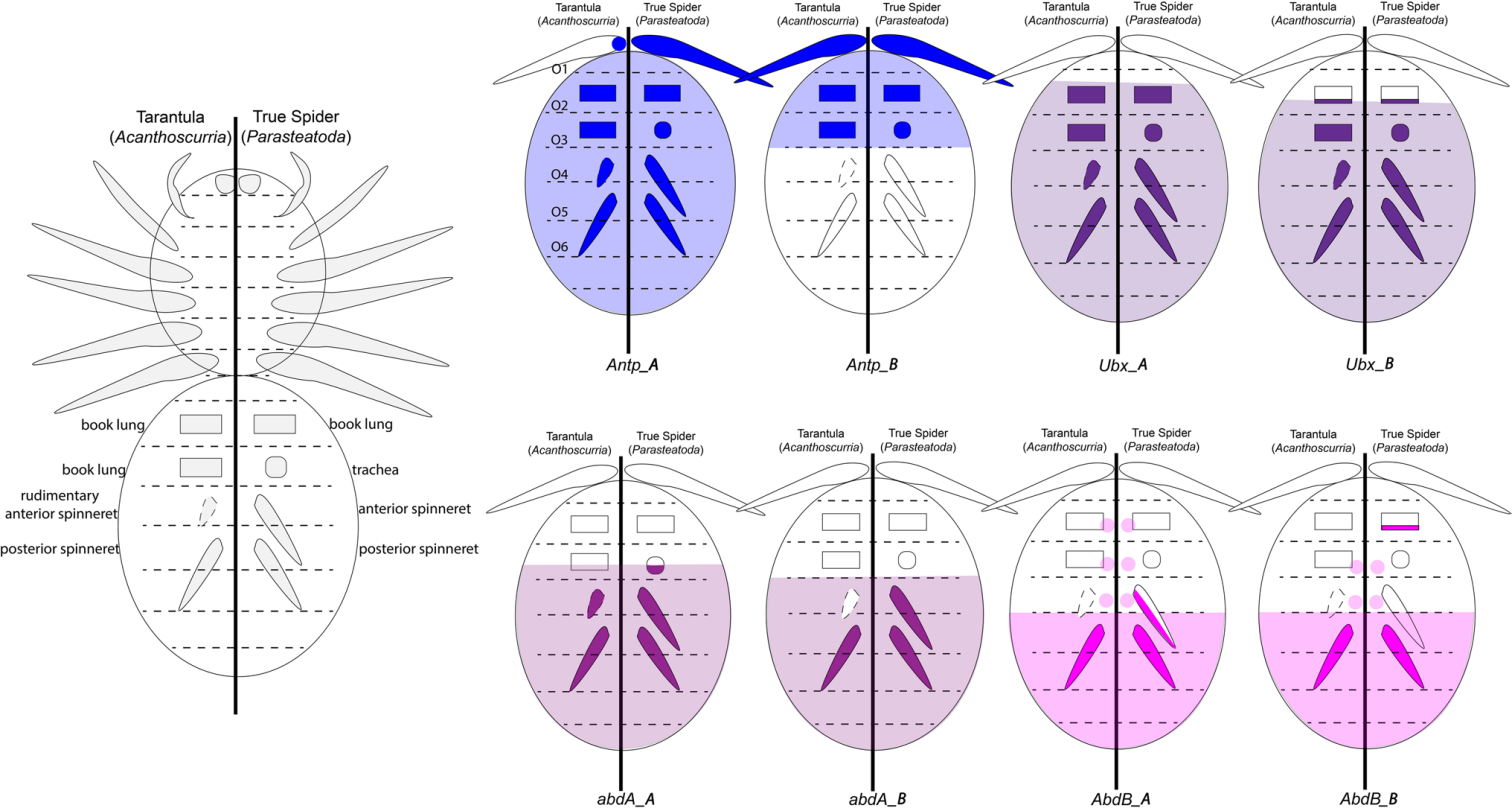
Summary and comparison of expression patterns in *A. geniculata* and true spiders (exemplified by expression in *P. tepidariorum*). Expression in the appendages is shown in stronger colors. Dot-like expression of *AbdB* near the indicated midline represents expression in the ventral nervous system; note that this expression extends more toward anterior than the classic Hox expression domain

Another potentially interesting tarantula-specific feature of *abdA* expression concerns the posterior book lungs (O3) that do not express *abdA_A* (Figs. 4 and 7). In true spiders, however, this segment forms trachea instead of book lungs, and *abdA_A* is expressed in the developing tracheal buds (Fig. 7 and Supplementary File 5) (Damen et al. 1998, Schwager et al. 2017). AbdA_A could thus repress book lung development and instead regulate tracheal development in this segment, a function as “modifier” of limb development that would in principle be consistent with its function in other arthropods (e.g., Konopova and Akam 2014; Martin et al. 2016). If this is the case, however, then this function of *abdA* is not conserved among chelicerates because, in scorpions, book lungs form in the presence of the single *abdA* gene (Sharma et al. 2014a).

### Future perspectives: investigation of spider Hox gene function in the opisthosoma

It is well known that a change in the Hox gene expression domain, a change in the biochemical function of a certain Hox gene (e.g., binding of new co-factors), or the regulation of downstream target genes is involved in the regulation of segment morphology (reviewed in Hughes and Kaufman 2002a). A recent analysis in crustaceans revealed that also combinatorial input of Hox genes is involved in specifying different appendage types (Alberstat et al. 2022).

Only the comprehensive functional analysis of Hox genes in spiders will allow us to understand how spider-specific morphological traits such as the spinnerets are specified during embryonic development. In Arachnopulmonata (e.g., scorpions, spiders), a potential WGD led to the duplication of many developmental genes, including most Hox genes, (e.g., Schwager et al. 2017). It is well known that such gene duplication events can result in neo- and/or sub-functionalization of the gene duplicate, which in turn might facilitate the establishment of novel morphological traits (reviewed in Taylor and Raes 2004), especially that the duplication and sub-functionalization of Hox genes might be involved in the evolution and diversification of new appendage types (such as spinnerets) in arachnopulmonate species. As duplicated genes might have partially redundant functions, it will be necessary to study the functions of both paralogs at the same time. Functional studies in the spider *P. tepidariorum* already showed that the double knockdown of Hox genes is challenging but feasible (e.g., Khadjeh et al. 2012). In the future, RNAi experiments in combination with the establishment of new functional tools (like CRISPR/Cas9-mediated gene knockout) will allow us to get a better insight into the question of how Hox genes influence the morphology of appendage-bearing opisthosomal segments in spiders.

### Supplementary information

ESM 1 (MSAP 60 kb)

ESM 2 (NEX 38 kb)

ESM 3 (DOCX 18 kb)

ESM 4 (PNG 4594 kb)

High resolution image (TIF 135375 kb)

ESM 5 (PNG 4822 kb)

High resolution image (TIF 132861 kb)

ESM 6 (PNG 4744 kb)

High resolution image (TIF 22741 kb)

ESM 7 (PNG 4850 kb)

High resolution image (TIF 133599 kb)

## Data Availability

All data generated or analyzed during this study are included in this published article and its supplementary information files.
