# Supplementary material for: Expression of posterior Hox genes and opisthosomal appendage development in a mygalomorph spider
Source: Dev Genes Evol. 2023 Jul 27;233(2):107–21. doi: 10.1007/s00427-023-00707-9 (PMC10746769; doi:10.1007/s00427-023-00707-9)
Supplement: Supplementary file 3 — (DOCX 18 kb) [file 427_2023_707_MOESM3_ESM.docx]

Sequence Identifiers

Ag-Antp_A TRINITY_DN75690_c2_g1_i2

Ag-Antp_B TRINITY_DN66381_c0_g2_i2; TRINITY_DN43429_c0_g1_i1

Ag-Ubx_A TRINITY_DN82983_c3_g1_i4

Ag-Ubx_B TRINITY_DN59883_c0_g1_i1

Ag-abdA_A TRINITY_DN79526_c1_g2_i6

Ag-abdA_B TRINITY_DN28902_c0_g1_i1; TRINITY_DN37887_c0_g1_i1

Ag-AbdB_A TRINITY_DN77332_c0_g2_i1

Ag-AbdB_B TRINITY_DN47292_c0_g1_i1

Cs-Antp_A CAA07499.1

Cs-Antp_B Locus_50289_Transcript_7/11

Cs-Ubx_A CAA07500.1

Cs-Ubx_B CAA07501.1

Cs-abdA_B Locus_2026_Transcript_2/2

Cs-abdA_A CAA07502.1

Cs-AbdB_A Locus_5470_Transcript_1/1

Cs-AbdB_B CAB40807.1

Pp-Antp_A c103414_g2_i7

Pp-Antp_B c105467_g2_i2

Pp-Ubx_A c109150_g2_i4

Pp-Ubx_B c111834_g6_i1

Pp-abdA_A c95590_g1_i1

Pp-abdA_B c104286_g1_i4

Pp-AbdB_A c106591_g2_i1

Pp-AbdB_B c97115_g1_i1

Pt-Antp_A NP_001310750.1

Pt-Antp_B XP_015917915.1

Pt-Ubx_A BBD75278.1

Pt-Ubx_B XP_021004342.1

Pt-abdA_A XP_015922000.1

Pt-abdA_B XP_015930560.1

Pt-AbdB_A XP_015930590.1

Pt-AbdB_B XP_015930562.1

Pt-ftz BBD75253.1

Complete Sequences of Ag-Antp2 and Ag-abdA2

Ag-Antp_A_DNA

AGTCCATACGCAGCCCAATGGGGAATTATGTGATCAATCCCAACGTCAGTATATTCAACCCCAGTATGCCTCTTCGCCTGCTCAAGGTGCGACATATCCCAGATTCCCCCCTTATGACAGACTAGAAATTCGACCTATCACTTCTTCACCGAATTCTCCAAGTCCTCCTGGCCAGTACTATGGCAATCCATGCAACCGAGCACCGCAAGGACCTCCTCTACCTCAGCCAGCTCACCAACCACCACAACATGCCCCTATGAGTCATCCTAACGCTTATGTACCTCAGGATGGACAAAACTGCCGAGGATCACCTCAGGATCCTAACGGACAACACGGAAGCTTGTCCCCAGCTCAGTATCCTAGTTGCAAGATGCAACCTCAACCTCCACCGCCACCTCACCCGCCCCCGCAACACCAGCAGCCACAAATGGGTCACGACCCCAGCGCAGTTGTTCATCGACAGGTAAATTCGGAATGTCCACCAGCAAACAGCATGCCCCCGTGCCAAAGCCCGGTTCATTCTCCTCAGCAACCTCAGATGTATCCTCAACAAAACCATAACGGACCCCCACCTAATCAGCAGCCAGTTCAACAACAGCCTGTTCAACAACCACCACCACCAGGAGGGAACATGCCCAGTCCACTCTACCCTTGGATGAGGAGTCAGTTTGAGAGGAAACGTGGCAGACAAACTTATACTCGTTACCAGACATTGGAACTGGAGAAGGAGTTCCATTTCAACAGGTACCTGACTCGAAGAAGAAGGATTGAAATAGCTCATGCTCTATGTCTGACGGAGAGGCAGATCAAAATAA

Ag-Antp_B_protein

VHTQPNGELCDQSQRQYIQPQYASSPAQGATYPRFPPYDRLEIRPITSSPNSPSPPGQYYGNPCNRAPQGPPLPQPAHQPPQHAPMSHPNAYVPQDGQNCRGSPQDPNGQHGSLSPAQYPSCKMQPQPPPPPHPPPQHQQPQMGHDPSAVVHRQVNSECPPANSMPPCQSPVHSPQQPQMYPQQNHNGPPPNQQPVQQQPVQQPPPPGGNMPSPLYPWMRSQFERKRGRQTYTRYQTLELEKEFHFNRYLTRRRRIEIAHALCLTERQIKI

Ag-abdA_B_DNA

CCGAATTACCCTCACTAAAGGGACTAGTCCTGCAGGTTTAAACGAATTCGCCCTTCAGGATCAACAGCAGCTATAATGGCCCCTTCTCAGTTCTTCCCTCATCCCGCCAGCGCTGATCTAGGTTCGAGCCTCACTTCATGCTCACAGCTCGGCATGCGGCAGCTGGAAGCCATACCTGACGTACCAAGATATCCGCGGATGTCCATTGCTGGACCAAACGGGTGTCCCAGGAGGAGAGGTCGGCAGACTTACACTCGATTCCAGACTCTGGAGTTGGAGAAAGAGTTTCACTTCAATCATTACCTGACACGGCGGAGGAGGATTGAAATAGCACACGCCCTGTGCCTCACCGAACGACAGATCAAGATCTGGTTCCAGAATAGGCGCATGAAGCTAAAAAAGGAAATGCGTGCCGTCAAAGAGATCAACGAGCAGGCTCGTATGGAGTCTAAATCCAAGGACGGTAGTGACAAGGACAAAAACAGTGGCGACGACACCAACACCACAGCTATCAACAAGAGCGTTAAAGAGGAGAGGAAAGTCTCTGAGCAGATATCCACCAACAGTGCTGTCCTCAACTCACCTGGTTTGGTCGTTGAAGACTGCAAGGTTCGCAGTTCTCAGACCTAACCAGACAGAACGTTCCAGATCAGCCATTTCATCTGACAGTGTGTCTCGGGATCTGCTCATTAAAATCACATGAGACTCCCATCGATCACAAGTGAAAAACGTGTTGTAATTTTTGTTTCTTCGTTATGCATTGTTTCCAAGTCCACTATAGATGCGTGCAATTGACTAGTGTTGCTAGCAACTGAAGCAAAACAGCGTTATACGTAATAGCAAGGGCGAATTCGCGGCCGCTAAATTCAATTCGCCCTATAGTGAGTCGTATTACAATTCACTGGCCGTCGTTTTACAACGTCGTGACTGGGAAAACCCTGGCGTTACCCAACTTAATCGCCTTGCAGCACATCCCCCTTTCGCCAGCTGGCGTAATAGCGAAGAGGCCCGCACCGATCGCCCTTCCCAACAGTTGCGCAGCCTATACGTACGGCAGTTTAAGGTTACACCTATAAAAGAGAGAGCCGTTATCGTCTGTTTGTGGATGTACGA

Ag-abdA_B_protein

MAPSQFFPHPASADLGSSLTSCSQLGMRQLEAIPDVPRYPRMSIAGPNGCPRRRGRQTYTRFQTLELEKEFHFNHYLTRRRRIEIAHALCLTERQIKIWFQNRRMKLKKEMRAVKEINEQARMESKSKDGSDKDKNSGDDTNTTAINKSVKEERKVSEQISTNSAVLNSPGLVVEDCKVRSSQT-

Primer Sequences

Ag-Antp_A_fw1 TCGAAATGGAGGTGGAGAAC

Ag-Antp_A_fw2 AACAACCACAGCAGCAGCAA

Ag-Antp_A_bw1 TATGATGAGCGAGTTCAGGT

Ag-Antp_A_bw2 CCCATTATGAGACCCGATTC

Ag-Antp_B_fw1 ACTTACGGAGTAGCGGTGAA

Ag-Antp_B_fw2 AGTCCATACGCAGCCCAATG

Ag-Antp_B_bw1 GTTTGGCTTTGTTCTCCTTC

Ag-Antp_B_bw2 TATTTTGATCTGCCTCTCCG

Ag-Ubx_A_fw1 CGAACAGAGCGGTTTCTACA

Ag-Ubx_A_fw2 CCACAGATCAGCCGTATAGA

Ag-Ubx_A_bw1 TTACGTTTTGGCATCGACCG

Ag-Ubx_A_bw2 ATAGCGGTGGTCTTGGTCGT

Ag-Ubx_B_fw1 CGAGCAACCCTACCGATTTC

Ag-Ubx_B_fw2 CAAGGACTGCTCGTACCCC

Ag-Ubx_B_bw1 TCCATACGTTTTTGCAGCCG

Ag-Ubx_B_bw2 CGACTGTTGTTGAGACTTCT

Ag-abdA_A_fw1 TTATGGACACGTTTTTGGGG

Ag-abdA_A_fw2 ATGCCGTATCATCGCTGTCA

Ag-abdA_A_bw1 TTATGGCAAACTCGCTTCGT

Ag-abdA_A_bw2 GTCTGAGCTGGGTATTACAG

Ag-abdA_B_fw1 TGGCTACTCCTTCAACTTGC

Ag-abdA_B_fw2 CCTTCTCAGTTCTTCCCTCA

Ag-abdA_B_bw1 TTTAATGAGCAGATCCCGAG

Ag-abdA_B_bw2 GATGAAATGGCTGATCTGGA

Ag-AbdB_A_fw1 GTCTGAGCTGGGTATTACAG

Ag-AbdB_A_fw2 TCGAAGTCGTTCAGCCATCT

Ag-AbdB_A_bw1 TTACTTCACTGCTGTTGTCG

Ag-AbdB_A_bw2 TCTGTCCGTTAGTAGCAGCA

Ag-AbdB_B_fw1 CGTGGTGTTGATCCAGCGTCT

Ag-AbdB_B_bw1 GGAGGATCTATGTGTCCATG

Ag-AbdB_B_bw2 CCAGAGTACGGCGTATAGTT

Cs_abdA_A_fw1 CACCCTTTACTACACCTCCA

Cs_abdA_A_fw1 GATACAACTCGACGGCTCAA

Cs_abdA_A_bw1 GCTTCTTTGTGTCAGGTTTC

Cs_abdA_A_bw2 GTAACTGAACAGAACGTGAG

Pp_abdA_A_fw1 GTTGAGCCCTAGTACCCATT

Pp_abdA_A_fw2 ATCTCAAACCACCCTTCAAC

Pp_abdA_A_bw1 GCACCCCCGATTTAACACAT

Pp_abdA_A_bw2 CGACCGTGTGCAAAATTGAT

Pp_abdA_B_fw1 TCGAACACGTTTATGGACAG

Pp_abdA_B_fw2 ATAGCAAGTACGCCAGTGAC

Pp_abdA_B_bw1 CAATATCAAGATCGCAGCAG

Pp_abdA_B_bw2 TGCTAAACATTCCTAGACCC
