# Supplementary figures and images for: Expression of posterior Hox genes and opisthosomal appendage development in a mygalomorph spider
Source: Dev Genes Evol. 2023 Jul 27;233(2):107–21. doi: 10.1007/s00427-023-00707-9 (PMC10746769; doi:10.1007/s00427-023-00707-9)

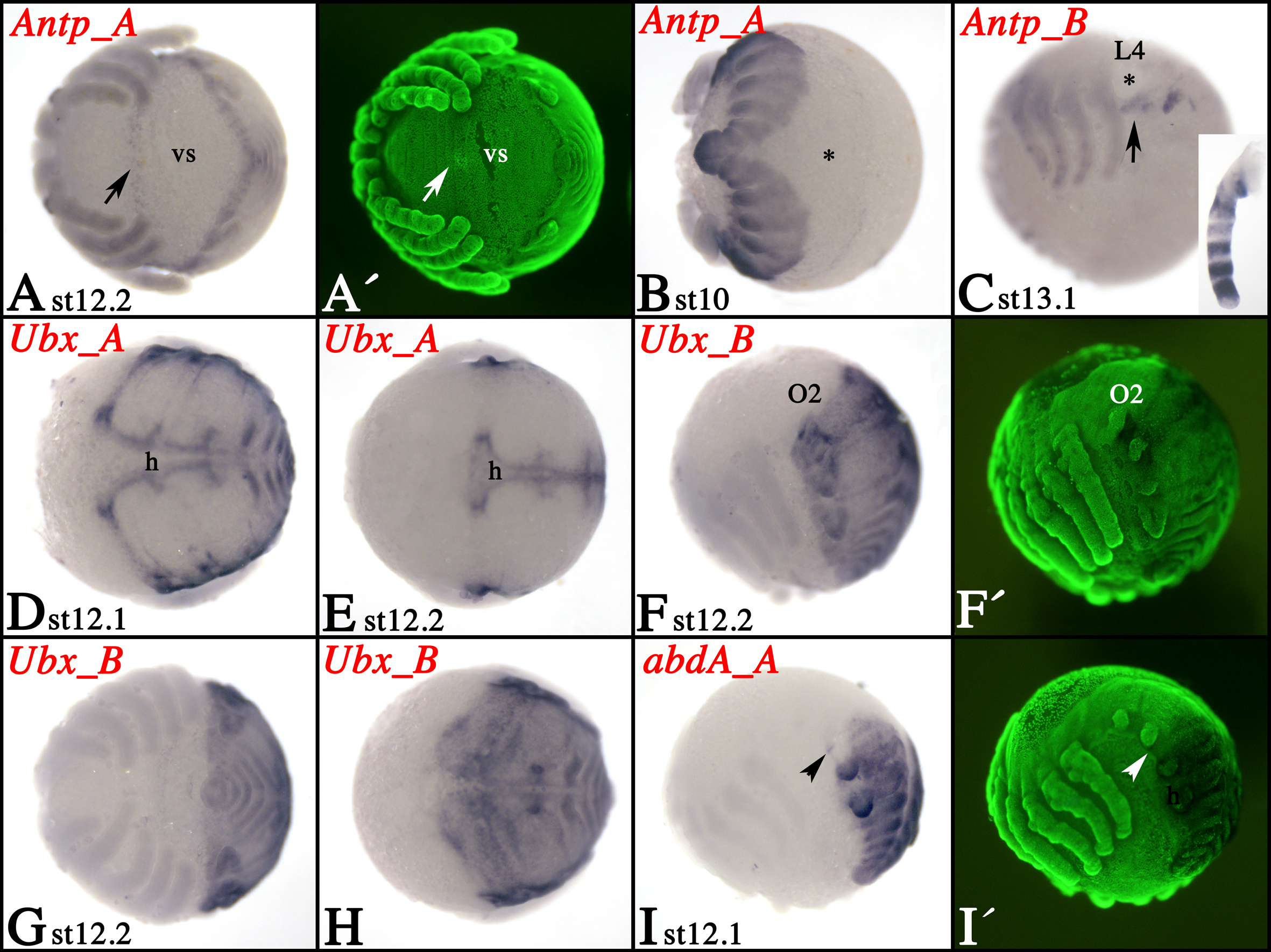

Supplement: Supplementary file 4 — (PNG 4594 kb) [file 427_2023_707_Fig8_ESM.png]

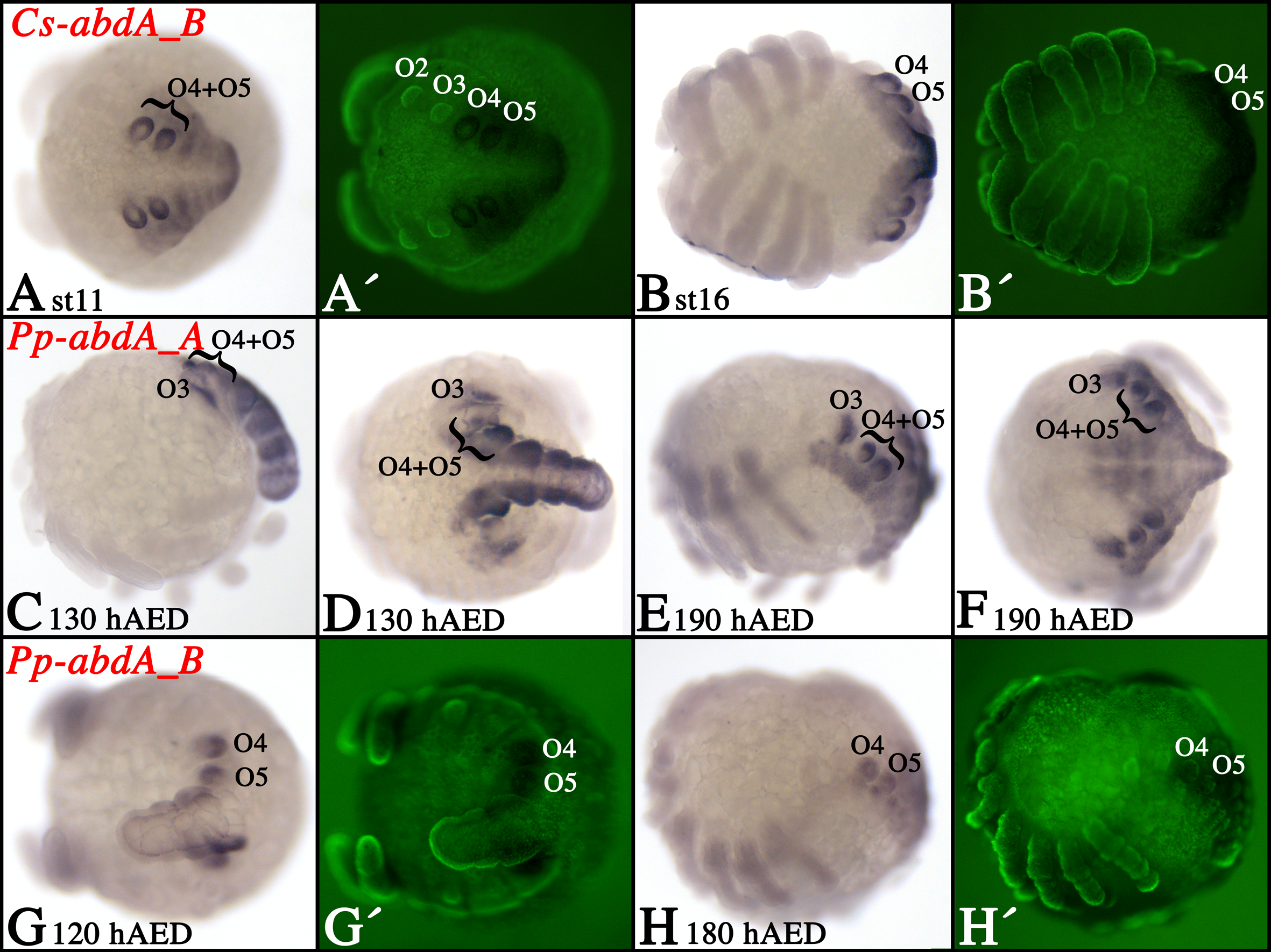

Supplement: Supplementary file 6 — (PNG 4822 kb) [file 427_2023_707_Fig9_ESM.png]

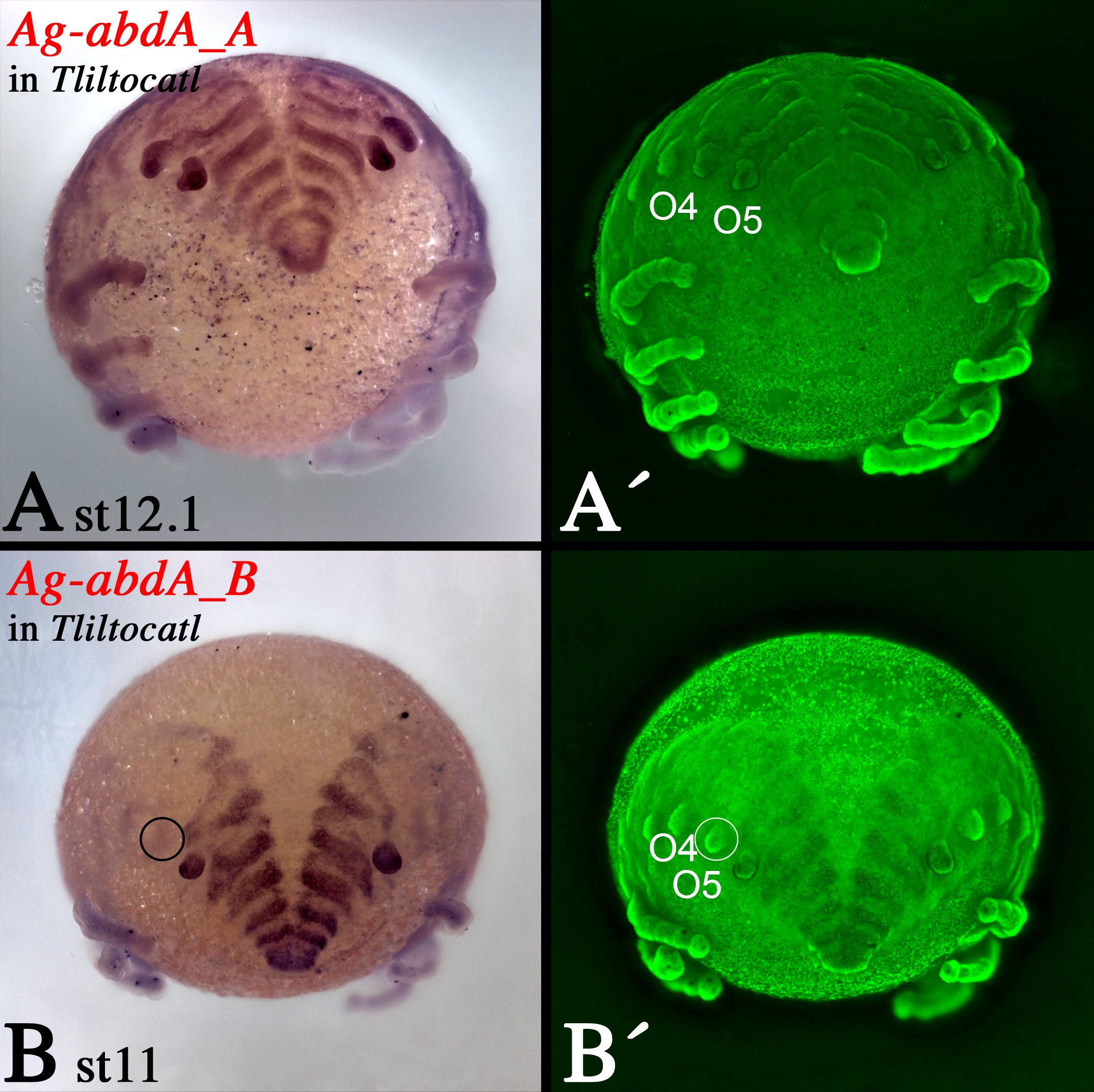

Supplement: Supplementary file 8 — (PNG 4744 kb) [file 427_2023_707_Fig10_ESM.png]

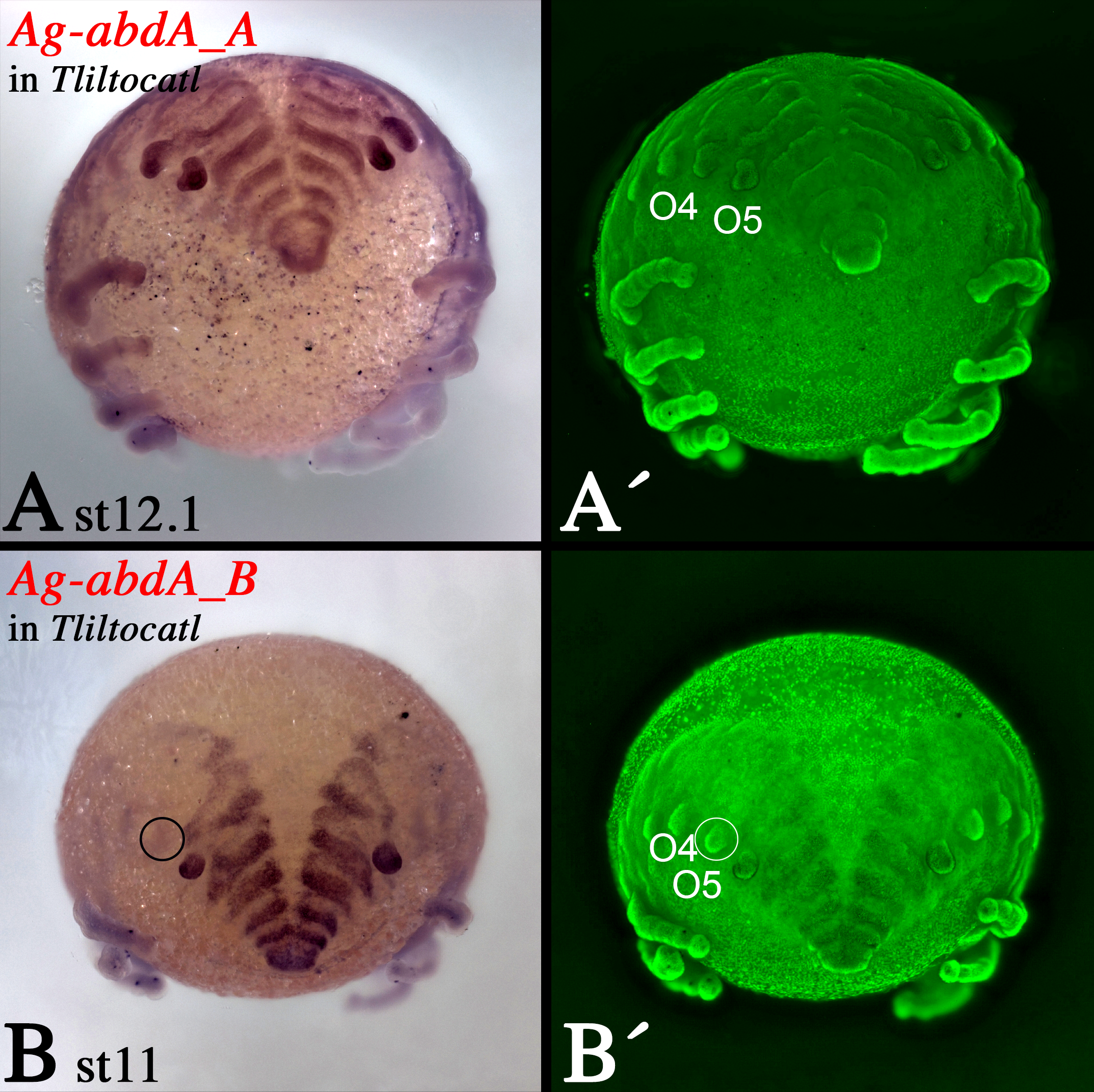

Supplement: Supplementary file 9 — High resolution image (TIF 22741 kb) [file 427_2023_707_MOESM6_ESM.tif]

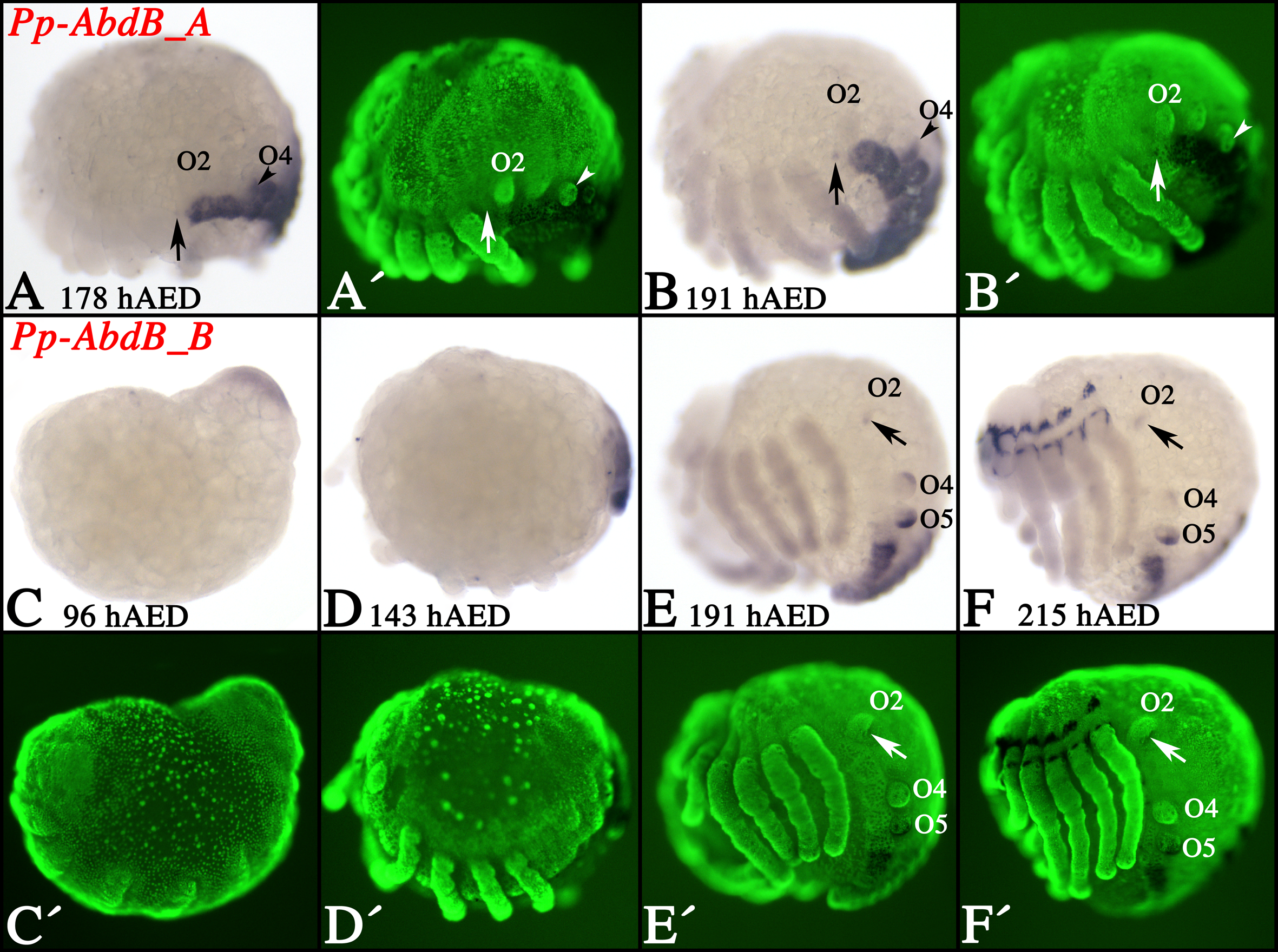

Supplement: Supplementary file 10 — (PNG 4850 kb) [file 427_2023_707_Fig11_ESM.png]
